# Prevention of Transcriptional *γ-globin* Gene Silencing by Inducing The Hereditary Persistence of Fetal Hemoglobin Point Mutation Using Chimeraplast-Mediated Gene Targeting 

**DOI:** 10.22074/cellj.2018.5181

**Published:** 2018-05-28

**Authors:** Reza Ranjbaran, Mahin Nikogoftar Zarif, Sedigheh Sharifzadeh, Habibollah Golafshan, Ali Akbar Pourfathollah

**Affiliations:** 1 Blood Transfusion Research Center, High Institute for Research and Education in Transfusion Medicine, Tehran, Iran; 2 Diagnostic Laboratory Sciences and Technology Research Center, School of Paramedical Sciences, Shiraz University of Medical Sciences, Shiraz, Iran

**Keywords:** Erythroid Progenitor Cells, Gene Silencing, Oligonucleotide-Directed Mutagenesis

## Abstract

**Objective:**

Hemoglobin F (HbF) augmentation is considered a clinically beneficial phenomenon in β-hemoglobinopathies. Prevention of γ-globin gene silencing, inspired by the hereditary persistence of fetal hemoglobin, may be a suitable strategy to upregulate HbF expression in these patients. Therefore, our objective was to assess the potential feasibility of induced *-117 G→A* substitution in *HBG* promoter in prevention of transcriptional silencing of the *γ-globin*.

**Materials and Methods:**

In this experimental study, human peripheral blood-derived hematopoietic stem cells (HSCs) and the K562 cell line were differentiated to erythroid cells. Erythroid maturation was examined using cell morphology parameters and flow cytometry analysis of CD235a expression. A synthesised chimeraplast was transfected to differentiating cells. The efficiency of chimeraplast delivery into target cells was assessed by flow cytometry. Restriction-fragment length polymorphism and DNA sequencing verified oligonucleotide-directed mutagenesis. Gene conversion frequency and globin genes expression was quantified through Allele specific-quantitaive polymerase chain reaction (AS-qPCR) and quantitative-PCR respectively.

**Results:**

Increase in CD235a-expressing cells along with observations made for different stages of erythroid maturation confirmed erythroid differentiation in HSCs and K562 cells. *γ* to *β-globin* gene switching was estimated to be on days 18-21 of HSC differentiation. Flow cytometry analysis showed that more than 70% of erythroid progenitor cells (EPCs) were transfected with the chimeraplast. The highest gene conversion efficiency was 7.2 and 11.1% in EPCs and K562 cells respectively. The induced mutation led to a 1.97-fold decrease in *β/γ-globin* gene expression in transfected EPCs at the experimental end point (day 28) whereas, due to the absence of *β-globin* gene expression following K562 differentiation, this rate was not evaluable.

**Conclusion:**

Our results suggest the effectiveness of chimeraplasty in induction of the mutation of interest in both EPCs and K562 cells. We also demonstrate that the single nucleotide promoter variant was able to significantly inhibit *γ-globin* gene silencing during erythroid differentiation.

## Introduction

Beta-thalassemia is one of the most common monogenic diseases, which turns out to be a significant public health concern due to its global burden and several complications associated with its homozygous form. Currently, thalassemia major patients are treated with regular blood transfusion, iron chelation and judicious splenectomy, all of which are temporary strategies accompanied by complications and life threatening side effects (1, 2).

Currently, allograft hematopoietic stem cell transplantation (HSCT) is thought to be the only definitive treatment for ß-thalassemia patients. Although, the success of transplantation, under ideal conditions, is more than 90%, the major limitation is lack of HLA-matched donors and therewith an outbreak of acute and chronic graft-versus-host disease (3). Several gene therapy techniques have been introduced which insert a normal copy of the *ß-globin* gene in ß-thalassemic patient’s hematopoietic stem cells (HSCs). These approaches have been undertaken in autologous HSCT to surmount the enormous problems that come along with allograft HSCT. The majority of gene therapy experiences are based on normal *ß-globin* gene integration into the genome of target cells through suitable vectors. Viral vectors such as oncoretroviruses and lentiviruses are common gene transfer agents in this context, which despite their high efficiency compared with other vectors, have limited practical use due to the risk of insertional mutagenesis and oncogenesis (4-6).

Chimeraplasty is a non-viral gene therapy technique in which single nucleotide conversion is executed via an artificial chimeric oligonucleotide. This oligonucleotide is synthesized from DNA and RNA fragments and is complementary to a specific sequence except for a single mismatch nucleotide at the substitution position of interest. Accordingly, this approach is exclusive to site- specific gene correction of point mutations (7-9).

Recently, hemoglobin F (HbF) inducers, both pharmaceutical and genetic agents, have attracted a vast interest for their potential therapeutic characteristics in ameliorating the severity of symptoms in Cooley’s anemia and sickle cell disease. Non-deletional hereditary persistence of fetal hemoglobin (HPFH) is a genetic disorder mainly caused by point mutations in the *γ-globin* gene promoter. These variants prevent *γ* to *ß-globin* gene switching during development. In some cases, HbF levels may reach up to 30% in heterozygotes and up to 100% in homozygous HPFH, and despite bearing such a genetic defect, HPFH is clinically asymptomatic even in homozygote form (10, 11).

Taking advantage of the HPFH genetic mechanism, we tried to induce the HPFH-like point mutation (*117 G→A*) in the *γ-globin* gene by applying the chimeraplasty approach. The main aim of this experimental study was therefore to prevent *γ-globin* gene silencing in erythroid progenitor cells (EPCs) and the erythroleukemia cell line K562 using gene therapy strategy appropriates for all ß-thalassemia cases. Subsequently, the consistency of *γ-globin* gene expression was tracked during erythroid differentiation. 

## Materials and Methods

### Chimeric oligonucleotide designing

In this experimental study, a 68-base synthesized chimeric RNA/DNA oligonucleotide (RDO) (now termed chimeraplast) was designed comprising a central core of 5 DNA-based nucleotides flanked by 10 2´-O-Methyl RNA sequences. To obtain stability in the chimeraplast structure, two nuclease-resistant hairpin caps of 4 T-residues were also designed followed by 25 complementary nucleotides to both the central DNA and the surrounding 2´-O-Methyl RNA sequences at the 5 end. Additionally, short regions with high melting temperature sequences were inserted at the 3´ end (9). The chimeraplast sequence was entirely matched to the corresponding genomic sequence of *HBG* promoter except for a single nucleotide change in the center of 5´base DNA stretch. This enabled the chimeraplast to form a mismatch with the G nucleotide located at position -117 of the *HBG* promoter. To evaluate the efficiency of transfection, an extra chimeric RDO was designed and labeled with FAM at its 5´ end with similar specification to the main chimeraplast. All experiments were carried out in duplicate for both treated and untreated control groups of HSCs and the K562 cell line.

### Erythroid differentiation 

Erythroid series were differentiated from HSCs existing in peripheral blood mononuclear cells (PBMNCs) using one-phase liquid medium culture system. Anti- coagulated whole blood samples, collected from normal volunteers with informed consent, were mixed with equal volume of phosphate buffered saline (PBS) and gently layered at a ratio of 3:1 onto the mononuclear separation medium (Lymphodex, Inno-Train, Germany). Following centrifugation, the mononuclear layer at the interface was harvested and cultured in a 6-well culture plate at a density of 6×10^6^ cells per ml of Iscove’s Modified Dulbecco’s Medium (IMDM, Caisson, USA) containing 30% fetal bovine serum (not heat-inactivated, Gibco, USA), 1% bovin serum albumin, ß-mercaptoethanol (10-5 M, Sigma, USA), dexamethasone sodium phosphate (10-6 M, Sigma, USA), human hollo-transferrin (0.3 mg/mL, Sigma, USA) and StemSpan™ Erythroid Expansion supplement which contained recombinant human stem cell factor (SCF), interleukin-3 (IL-3) and erythropoietin (EPO) (STEMCELL Technologies, Canada). 

Cells were then incubated for 28 days at 37°C with 5% CO_2_ and the differentiation medium was refreshed on day 14. At four time points over a 28-day period (days 7, 14, 21 and 28) cells were harvested from culture media and prepared for morphology assessment (Wright staining). Consequently, the expression of the surface marker, CD235a, was assessed by flow cytometry and the expression of globin gene was quantified using quantitative reverse transcription polymerase chain reaction (RT-qPCR). 

K562 cells (ATCC, USA) were also differentiated with analogous conditions in 7 days and were similarly assessed for erythroid differentiation as well as *globin* gene expression profile. 

### Flow cytometry

Erythroid differentiation was tracked by evaluating the percentage of CD235a (Glycophorin A) positive cells per well of each individual 6-well culture plate. Cells were harvested, transferred to a 1.5 ml microtube and spun down at 500 xg for 5 minutes. To prevent the interference of pre-existing red blood cells (RBCs), the cell pellet was washed in 1 ml of RBC lysing solution and incubated with 5 µl of monoclonal anti-Glycophorin A-phycoerythrin (PE) (Dako, Denmark). After, the cells were washed in PBS and the single cell suspension, prepared in 500 µl PBS, was subjected to a flow cytometer (BD FACSCalibur, USA) versus PE labeled isotype control. Data were analysed using the FlowJo 7.6 software (Tree Star Inc., USA).

### Cell transfection

Upon erythroid colony growth on day 16, following culture initiation, once erythroid cells were strikingly increased in number, cells were transfected with RDO using a polycationic vector, polyethyleneimine (PEI, Sigma, USA). PEI (30 µl of 1 mg/ml) and RDO (10 µg) had been previously diluted in 120 µl of serum and antibiotic free Opti-MEM media (Gibco, USA), and incubated at room temperature for 10 minutes to form RDO-PEI complexes. Subsequently, 850 µl of supplemented IMDM was added to the complex and the mixture was then added dropwise to 6-well plates and mixed by gently rocking. The same trend was performed for the K562 cell line with the transfection time being on day 1. 

### Transfection efficiency

To evaluate transfection efficiency, FAM-RDOtransfected EPCs and K562 cells were detected with flow cytometry and fluorescent microscopy on the second day of transfection. Cells were initially harvested and washed in PBS, and then assessed prior and after labeling with anti CD235a-PE antibody through the FL1 and FL2 channels of a BD FACSCalibur flow cytometer. Additionally, a number of cells were evaluated for nuclear entry of chimeric oligonucleotides by Fluorescence Microscope Axiostar Plus (Gottingen, Germany). 

### Polymerase chain reaction-restriction-fragment length polymorphism 

Presence of the point mutation in the *HBG* promoter resulted in a restriction site for Tru1l (MseI) restriction endonuclease. Genomic DNA was extracted from transfected cells and the region of interest was amplified using specific primers to amplify a 223bp DNA fragment (Table 1). The amplicon was subsequently digested with Tru1l restriction enzyme (Thermo scientific, Lithuania) for up to 16 hours according to manufacturers’instructions and DNA fragments were separated by electrophoresis on a 2% agarose gel. 

**Table 1:** Primer Sequences used for RT-qPCR, AS-qPCR, conventional PCR amplification and Sanger sequencing


Target	Sequence primer (5ˊ-3ˊ)

*β-actin* cDNA	F: ATCGTGCGTGACATTAAGGAG
	R: GAAGGAAGGCTGGAAGAGTG
*β-globin* cDNA	F: CTGAGGAGAAGTCTGCCGTTA
	R: AACAGCATCAGGAGTGGACA
*γ-globin* cDNA	F: TTCACAGAGGAGGACAAGGCTAC
	R: GCAGAGGCAGAGGACAGGTT
BCL11a-xl cDNA	F: GTCTCGCCGCAAGCAAGG
	R: GCCGTGGTCTGGTTCATCATCT
AS-qPCR wild type	F: AAACTGGAATGACTGAATCG
	R: CTTGTCAAGGCTATTGGGC
AS-qPCR mutant	F: AAACTGGAATGACTGAATCG
	R: CTTGTCAAGGCTATTGGGT
*γ-globin* promoter and sequencing primer	F: TTATTGATAACCTCAGACGTTCC
	R: ATCTCAATGCAAATATCTGTCTG


RT-qPCR; Quantitative reverse transcription polymerase chain reaction and AS-qPCR; Allele specific-quantitaive PCR.

### DNA sequencing

Digested fragments were isolated and extracted by GEL DNA recovery kit (Vivantis, Malaysia), and then ligated with T4 DNA ligase (Vivantis, Malaysia) for 4 hours at 16ºC. Eventually, ligated DNA was reamplified and directly sequenced along with undigested wild-type fragment using the same primer pairs used in PCR- restriction-fragment length polymorphism (PCR-RFLP) (Table 1).

### Chimeraplasty efficiency by allele specific-quantitative polymerase chain reaction

Allele-specific quantitative PCR (AS-qPCR) was carried out by using a real-time PCR system (QIAGEN, Germany) to quantify the relative allelic rate of mutant HBG promoter. Wild type and mutant HBG promoters were amplified by a single common forward primer and reverse allele-specific primers (Table 1). Amplification efficiency of the intended amplicons were determined by the standard curve of each allele through logarithmic dilution of PCR products of each amplicon. Eventually, the ratio of mutant alleles versus wild-type was quantified. Subsequently, amplified products were verified by 2% agarose gel electrophoresis.

### Quantitative reverse transcription polymerase chain reaction

Total RNA was purified from erythroid and K562 cells using the TRIzol reagent (Life Technologies, USA) and quantified using the NanoDrop spectrophotometer. Subsequently, DNaseI treated RNA was reverse transcribed to complementary DNA (cDNA) by using the one-step SYBR PrimeScript RT Reagent Kit (TaKaRa, Japan). Relative quantification of *ß* and *γ-globin* genes expression were assessed using specific primers and SYBR Green PCR mastermix (TaKaRa, Japan) (Table 1). Expression analysis of *ß-actin*, the housekeeping gene, was also evaluated during *in vitro* erythroid differentiation in transfected and non-transfected cells. Standard curves were plotted and amplification efficiency of each gene was obtained through dilution series of PCR amplicons and data were finally recorded as *ß/γ-globin* transcript expression ratio. All reactions were undertaken in duplicate.

### Statistical analysis 

Data are presented as mean ± SD based on replicate experiments. Independent t test was used to compare unpaired groups. The results with P<0.05 was considered as statistically significant. All data were statistically analysed and visualized using the GraphPad Prism software (version 6.04, GraphPad Software, CA).

## Results

### Erythroid differentiation of peripheral blood hematopoietic stem cells and the K562 cell line

**Fig.1:**
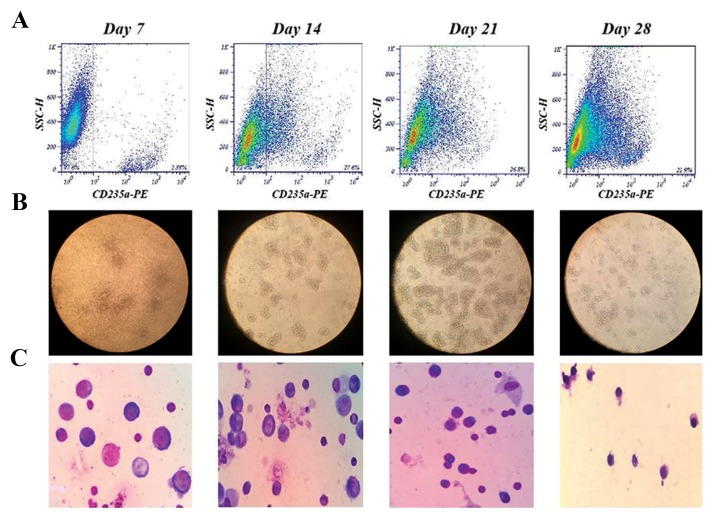
Verification of erythroid differentiation by expression analysis of CD235a, inverted and light (×100) microscopic assays. A. After 28 days of hematopoietic progenitor cells erythroid differentiation, the percentage of CD235a positive cells changed from 2.38% on day 7 to an average of 27.6, 26.8 and 21.9% on days 14, 21 and 28 respectively, B. Erythroid colonies expanded and gradually spread during a 28-day time period, and C. Between days 7 and 14, cells displayed pronormoblast and basophilic normoblast morphology. On day 21, a considerable number of erythroid cells transformed into polychromatophilic normoblast, which were then differentiated into orthochromatophilic normoblast by the end of day 28.

HSCs from PBMNCs were successfully differentiated to mature erythroid cells. Flow cytometry assessment revealed an approximately two-fold increase in the percentage of CD235a positive cells (1.4 to 2.38%) after one-week of differentiation. Two weeks after differentiation, CD235a positive cells reached the highest value of 27.6% and remained rather constant (26.8%) within the next 7 day interval even though a slight decrease was observed at the experimental end point (21.9%) (Fig .1A).

The first colonies of erythroid series were discernible under the inverted microscope 3 days following the culture initiation. Between days 7 and 14, cells displayed early normoblast morphology (pronormoblast and basophilic normoblast) with Wright’s stain. Colonies expanded and gradually spread over a period of 21 days. On day 21, a considerable number of erythroid cells transformed into polychromatophilic normoblasts, which gave rise to orthochromatophilic normoblasts on day 28 (Fig .1B, C).

Different stages of erythroid maturation in growth factor-stimulated K562 cells were also observed with Romanowsky stain. After 7 days of treatment with erythroid differentiation factors, K562 cells differentiated into orthochromatophilic normoblasts and the percentage of CD235 positive cells changed from 3.26% on day 0 to an average of 42.2% on day 7.

### Transfection efficiency

**Fig.2:**
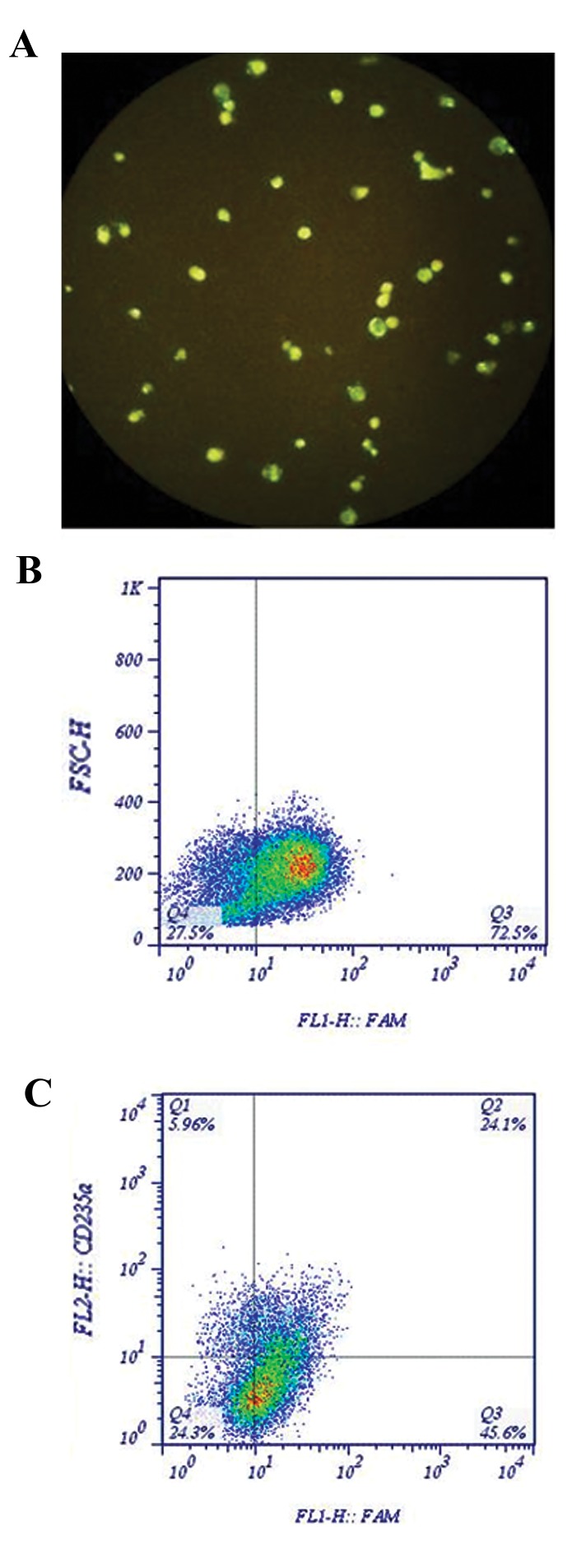
Efficiency of RDO transfection in erythroid progenitor cells (EPCs). A. Transfection efficiency was assessed through fluorescent microscope, B. Flow cytometry 48 hours post-transfection. The dot-plot histogram represents FAM-labeled RDO uptake by more than 70% of cells, and C. Quadrant regions display the percentage of transfected EPCs (double positive population).

After 24 and 48 hours, the percentage of transfected cells including CD235a-positive cell population was found to be more than 70% (ranging from 70 to 80%) by flow cytometry assessment. However, the green fluorescent signal visualized from FAM-labeled RDOs in nuclei showed that only one third of the cells were successfully nucleofected at the final time point (Fig .2). Nucleofection was assessed after adequate resting time including 24 and 48 hours following transfection. Nevertheless, it might be assumed that even after 2 days, RDOs could still introduce into the cells, leading to an elevation of nucleofection efficiency. 

### Polymerase chain reaction- restriction-fragment length polymorphism

**Fig.3:**
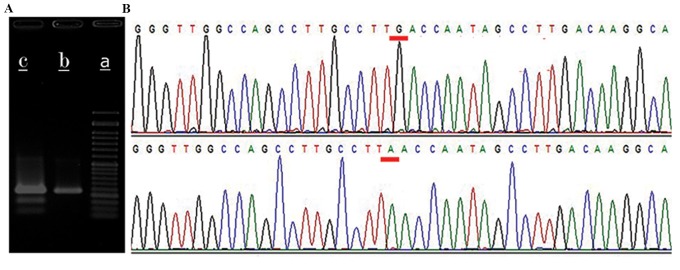
Confirmation of the presence of induced point mutation by PCR-RFLPand Sanger sequencing methods. A. Visual representation of restriction endonuclease digestion of the 223 bp fragment of the HBG promoter. (a) 50 bp ladder, PCR-amplified and Tru1l-treated HBG promoter from (b) untreated and (c) RDO-treated EPCs and B. The sequence of digested 151 bp and 72 bpfragments following gel purification, ligation and reamplification (A allele) andundigested 223 bp fragment of the HBG promoter (G allele).

A 223 bp DNA fragment spanning the target region in *HBG* promoter was amplified in transfected and non-transfected cells with specific primers (Table 1), and then incubated with the Tru1l restriction enzyme. Products showed the co-existence of the undigested 223 bp and the two digested 151 bp and 72 bp fragments representing the partially expected nucleotide substitution through chimeraplasty in both EPCs and the K562 cell line (Fig .3).

### DNA sequencing

Gel-extracted 151 bp and 72 bp fragments were ligated, re-amplified and sequenced with the Sanger method, confirming the PCR-RFLP result by showing the G→A substitution at position -117 in the *HBG* promoter (Fig .3). 

### Conversion efficiency 

**Fig.4:**
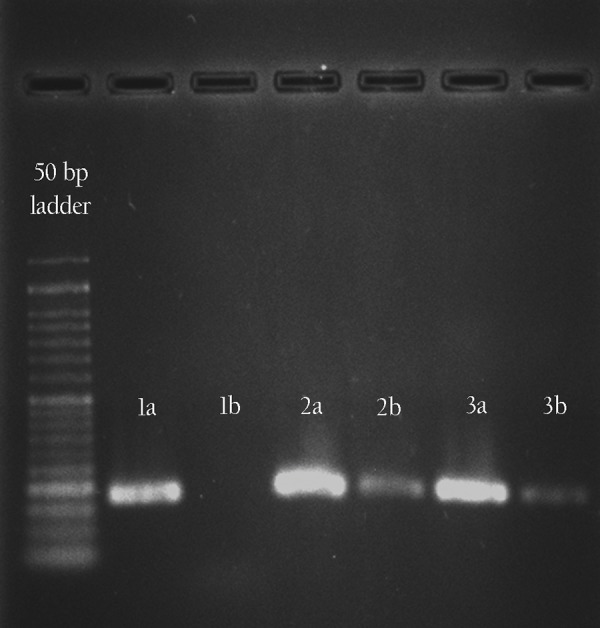
Agarose gel electrophoresis of AS-qPCR products amplified in 1) untreated EPCs, 2) treated K562 and 3) treated EPC samples with G-allele (a) and A-allele (b) specific primers.

To assess the quantification of gene conversion, AS-qPCRwas used. Amplification efficiency (E) was determined to be 0.98 for the wild-type allele, and 1.0 for the mutant alleleand the housekeeping gene. The mean cycle of threshold(CT) value of mutant (A) allele, non-mutant (G) allele and *ß-actin* were measured in untreated and treated EPCs on day19 (three days after transfection) in duplicate. The resultsshowed no amplification for the mutant allele in untreated samples. The allelic ratio of mutant *HBG* promoter towild type in each sample was calculated based on *A-allele* efficiency ^CT^/(*G-allele* efficiency ^CT+^*A-allele* efficiency^CT^)×100. Accordingly, the efficiency of gene conversionin EPCs was quantified as 5.9% and 7.2% in 2 successfulexperiments while this value was measured to be 11.1% in the K562 cell line. Specific amplification of each allele wasverified by agarose gel electrophoresis which resulted in one specific band of 172 bp (Fig .4). 

### *Gamma* and *ß-globin* gene transcript levels

Adecreasing trend in the ratio of *ß/γ globin* gene expressionlevel was observed from day 0 to day 18 during erythroid differentiation in the untreated control group. This parameter stayed at a relatively constant level amongst days 18 to 21 while an increase was observed from day 21 onward. Thesefindings suggest that *γ* to *ß-globin* gene switching initiatedaround day 21, concurrent with the polychromatophilic normoblast phase of maturation. Moreover, the high *ß/γ* expression rate in the first few experimental days (in which no switching had occurred and *ß-globin* expression was not expected) was likely due to the presenceof reticulocytes and the relative stability of *globin* transcripts in the culture environment. However, when the reticulocytes were destroyed overtime, this parameter got much closer to the expected value. 

Along with cell maturation, relative expression level of *γ-globin* gene in K562 cells showed an increase of approximately 12-fold (P<0.0001) on day 7 when A compared with the base line expression on day 0. However, the induction of differentiation did not trigger *ß-globin* gene expression and thus the related transcript overexpression could not be recognized using RT-qPCR prior and after K562 cell differentiation.

### Effect of the inducible variant on the expression level of *γ* and *ß-globin*

Subsequent to nucleotide substitution in the genomicregion of interest, expression levels of *ß* and *γ-globin* were measured at the transcript level in the treated cells, which were previously subjected to erythroid differentiation. However, due to undetectable levels of *ß-globin* transcriptsin either normal or mutant K562 cells, it was not possible to compare the relative rate of*ß* and *γ-globin* transcript levels. 

Evaluation of *γ* and *ß-globin* expression patterns in transfected EPCs during erythroid differentiation showedthat, in comparison with the untreated group, there was a1.51-fold increase (P<0.05) in *γ/ß-globin* expression ratio onday 21 in treated cells and the rate of change rose to 1.97-fold increase (P<0.05) at the experimental end point (day 28). 

**Fig.5:**
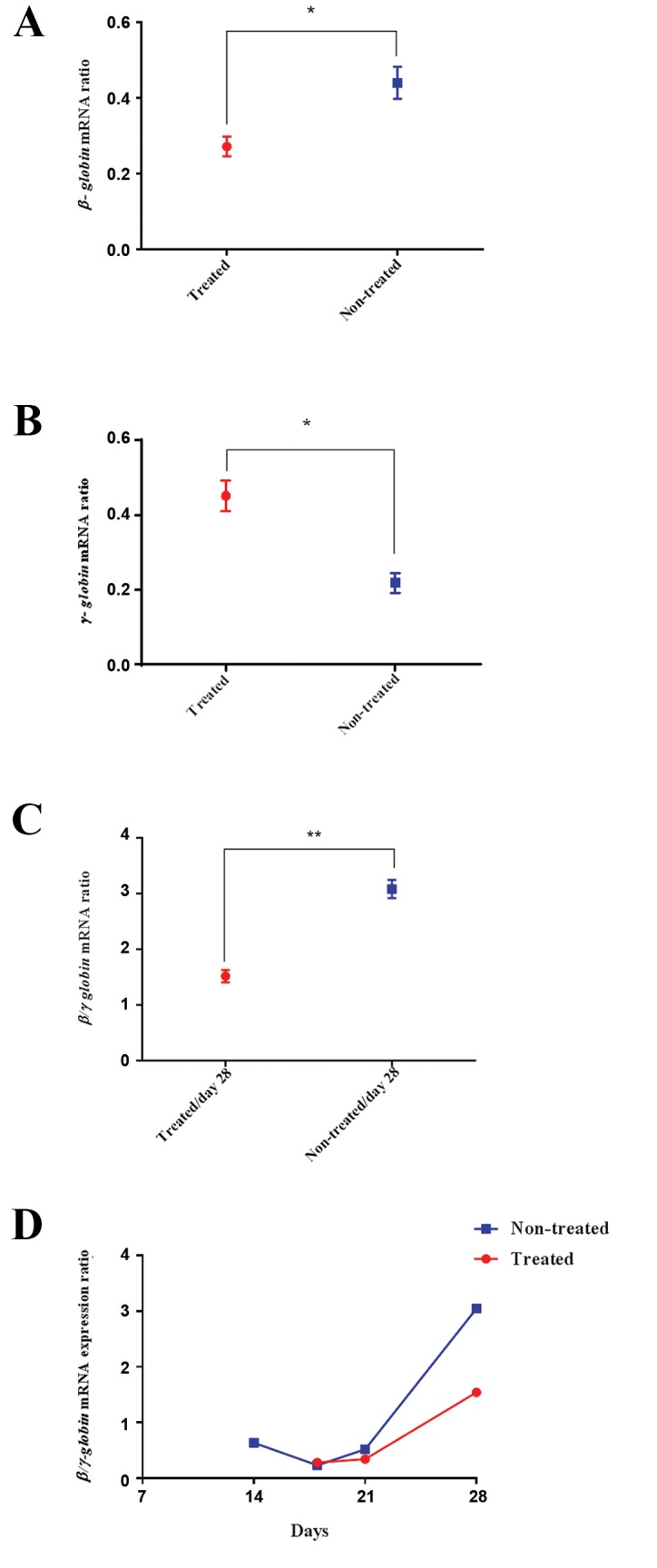
Expression analysis of *ß-globin, γ-globin* and *ß/γ-globin* transcript ratio. A. *ß-globin* expression in treated EPCs was down-regulated to 0.59- fold (*; P<0.05) and B. *γ-globin* gene expression showed 1.82-fold increase in treated EPCs (*; P<0.05) on the final day of differentiation when compared with non-treated cells. Representative *ß/γ-globin* transcript ratio in RDO-treated, and Non-treated EPCs, C. At day 28 (**; P<0.001) and D. During erythroid differentiation.

Accordingly, the relative expression of γ-globin to the housekeeping gene (*ß-actin*) was significantly higher in treated cells when compared with non-treated controls on day 28 (0.42 vs. 0.23, P<0.05). In contrast, *ß-globin* relative expression in treated cells was significantlydecreased in comparison with non-treated controls on the same day (0.27 vs. 0.46, P<0.05). Taken together, these results suggest the effectiveness of the inducible single nucleotide variant in significantly preventing *γ-globingene* silencing (Fig .5). However, there was no significant difference in *γ-globin* gene expression between treated and untreated K562 cells at different differentiation days.

## Discussion

Although the rate of success of chimeraplasty is still under debate, it has been so far used for inducing or modifying point mutation in various studies. This rate varies substantially in previous studies from 0.05% reported by Igoucheva et al. (12) to 50% reported by Cole-strauss et al. (13). Surveys have shown that numerous factors including RDO structure, quality, concentration and size along with type of cell and delivery system may significantly influence the success or conversion rate of this method (14). 

Here, we used chimeraplasty for a G→A nucleotide substitution at position -117 of the *γ-globin* gene promoter in EPCs originating from peripheral blood HSCs. Besides, we applied this method to K562 cells to set up the transfection, mutagenesis and erythroid differentiation. This specific cell line was used since it can spontaneously develop characteristics similar to EPCs and predominantly expresses the *γ-globin* gene.

Although our results showed a more efficient rate of nucleotide conversion in K562 cells in comparison with EPCs, in contrast to Addya et al. (15) and Isoda et al. (16), no *ß-globin* gene expression was detected in differentiated K562 cells. However, these findings corroborate previous observations where *ß-globin* expression was not observed in K562 cells (17, 18).

A number of investigations have reported that the inability of erythroid growth factors (IL-3, EPO and SCF) in mediating the *BCL11a-xl* (i.e. the main transcription factor for *γ* to *ß-globin* switching) signaling pathway may be the major reason that globin switching does not take place in K562 cells (19). We, however, observed that albeit *BCL11a-xl* expression was upregulated by 6-fold following erythroid differentiation, *ß-globin* did not show any expression in K562 cells (data not shown). Some other studies have suggested that homologous recombination (HR) is a potential molecular mechanism underlying oligonucleotide-mediated site directed mutagenesis. HR comprises a series of molecular processes essential for DNA repair. Rad51 nucleoprotein and its homologue RecA in prokaryotes play a key role in HR reactions and their recombinase activity is required for efficient gene recombination (20, 21). There is enough evidence to show that Rad51 recombinase has a higher expression level in diverse cancer cells in comparison with normal cells. This phenomenon may indeed be the possible cause for the diversity in conversion efficiencies obtained. Likewise, it may also explain the difference in results obtained from the two types of cells used in this study where increased expression of Rad51 recombinase in K562 cell line has been recently shown (22, 23). Furthermore, a lower purity of directly differentiated HPCs at the transfection time, due to the one-phase medium liquid culture system, may also affect the efficiency of targeted mutagenesis in EPCs. 

In a recent similar work by Chin et al. (24) triplex-forming peptide nucleic acids were utilized to mediate targeted gene conversion of -117 HPFH and hypoxia response element (HRE) donor DNA in expansion conditions of CD34+ cells. This resulted in significant *γ-globin* gene upregulation that was mostly the consequence of the HRE element and hypoxic culture conditions rather than the HPFH variant since no considerable upregulation was observed for γ-globin gene by using only the HPFH donor DNA. This discrepancy can be explained in the following two ways. First, it has been recently found that the -117 G>A variant is associated with COUP-TFII DR-binding element disruption in the *γ-globin* gene promoter, resulting in stage-specific *γ-globin* gene silencing but not increased *γ-globin* gene expression in undifferentiated CD34+ cells (25, 26). This hypothesis is corroborated with our results where no significant difference in γ-globin gene expression was detected between treated and untreated K562 cells in which no *γ* to *ß-globin* gene switching had occurred after erythroid differentiation.

Secondly, if the effect of such variants results in elevated expression of *γ-globin* rather than preventing *γ-globin* gene inactivation, it may lead to an imbalance in the ratio of a and non-a (*ß+γ*) globin chain synthesis, which has not been previously observed in HPFH (27, 28). Therefore, we decided to work on differentiating cells undergoing *γ-globin* gene switching process. 

It is worth noting that in contrast to our study, which directly targeted the cell genome, Li et al. (29) designed a chimeric oligonucleotide to trigger gene conversion in a plasmid, however, it resulted in a lower efficiency associated with plasmid instability in subcloning cells. 

## Conclusion

In the present study, the *HBG* promoter inducible variant *(117 G→A)* was successfully introduced into the genome of EPCs through chimeraplasty and noticeably reduced *γ-globin* gene silencing. However, current laboratory approaches are not capable of elucidating the effects of γ-globin gene upregulation on either increasing the total hemoglobin or the clinical status of patients suffering from ß-hemoglobinopathies. Consequently, further investigations are warranted to introduce genetically manipulated cells to animal models. 
